# Effect of epidural spinal cord stimulation combined with personalized neurorehabilitation in chronic motor-complete spinal cord injury: study protocol for the phase II single-arm feasibility RESTORES (RESToration Of Rehabilitative function with Epidural spinal Stimulation) trial

**DOI:** 10.3389/fneur.2026.1967078

**Published:** 2026-10-09

**Authors:** Kai Rui Wan, Zhi Yan Valerie Ng, Seng Kwee Wee, Fatimah Misbaah, Wen Li Lui, Min Wee Phua, Tegan Plunkett, Neethu Robinson, Aung Aung Phyo Wai, Cuntai Guan, Ananda Sidarta, Rosa Qi Yue So, Brian Premchand, Kezia Susanto, Luca Cocchi, Seyed Ehsan Saffari, Rui Xin Justin Ker, Wai Hoe Ng

**Affiliations:** 1 Department of Neurosurgery, National Neuroscience Institute, Tan Tock Seng Hospital, Singapore, Singapore; 2 Department of Neurosurgery, National Neuroscience Institute, Singapore General Hospital, Singapore, Singapore; 3 Department of Rehabilitation Medicine, Tan Tock Seng Hospital, Singapore, Singapore; 4 Singapore Institute of Technology, Singapore, Singapore; 5 College of Computing and Data Science, Nanyang Technological University, Singapore, Singapore; 6 Rehabilitation Research Institute of Singapore, Nanyang Technological University, Singapore, Singapore; 7 Institute of Advanced Intelligence and Computing (IAIC), Agency for Science, Technology and Research (A*STAR), Singapore, Singapore; 8 Department of Biomedical Engineering, National University of Singapore, Singapore, Singapore; 9 Brain and Mental Health Program, QIMR Berghofer, Brisbane, QLD, Australia; 10 School of Biomedical Sciences, University of Queensland, Brisbane, QLD, Australia; 11 Department of Neurology, National Neuroscience Institute, Tan Tock Seng Hospital, Singapore, Singapore

**Keywords:** epidural spinal stimulation, motor recovery, neuromodulation, neuroplasticity, neurorehabiliation, spinal cord injury

## Abstract

**Background:**

Chronic motor-complete spinal cord injury (SCI) results in profound neurological disability with minimal prospects for functional recovery using conventional rehabilitation strategies. Emerging evidence suggests that epidural spinal cord stimulation (eSCS) can activate dormant spinal locomotor networks and restore volitional and autonomic functions when combined with intensive activity-based training. However, systematic evaluation of multimodal neuromodulation approaches integrating advanced rehabilitation and computational neurophysiology remains limited.

**Methods:**

The RESTORES trial (RESToration Of Rehabilitative function with Epidural spinal Stimulation) is a prospective, single-arm Phase II feasibility study enrolling 15 adults with chronic (≥1 year) motor-complete SCI (American Spinal Injury Association Impairment scale (AIS) A or B) between C5 and T12. Participants will undergo implantation of an epidural spinal cord stimulator followed by a 24-week program of structured stimulation-coupled personalised neurorehabilitation (five sessions per week). Clinical outcomes will be evaluated using standardized neurological and functional measures including the AIS motor score, Spinal Cord Independence Measure version III (SCIM-III), Walking Index for Spinal Cord Injury II (WISCI-II), and Trunk Assessment Scale (TASS). Autonomic function and quality of life will also be assessed. Multimodal neurophysiological measurements—including surface electromyography (sEMG), electroencephalography (EEG), resting-state functional magnetic resonance imaging (rs-fMRI), and computer-vision-based movement analysis—will be performed to investigate neural recovery mechanisms and inform the development of adaptive neuromodulation systems.

**Study outcomes:**

The primary outcome is the safety of epidural spinal cord stimulation implantation, defined as the absence of device explantation for safety-related reasons within 6 months following implantation. Secondary outcomes include changes in AIS motor score, functional outcomes (SCIM, WISCI-II, TASS), autonomic function, quality of life, and neurophysiological biomarkers.

**Discussion:**

This study integrates neuromodulation, advanced rehabilitation technologies, and multimodal computational neurophysiology to evaluate both clinical efficacy and neural mechanisms of recovery following chronic SCI. Findings from the RESTORES trial will provide critical safety and feasibility data to inform future multicenter trials and the development of closed-loop spinal cord stimulation systems.

**Clinical trial registration:**

ClinicalTrials.gov, Identifier NCT05644171.

## 1 Introduction

Spinal cord injury (SCI) results in severe and often permanent neurological deficits, leading to loss of voluntary motor control, autonomic dysfunction, and substantial long-term disability (1, 2). Individuals with motor-complete injuries (American Spinal Injury Association Impairment Scale [AIS] grades A–B) have a particularly poor prognosis for neurological recovery, with meaningful restoration of voluntary motor control after a clinically complete lesion considered extremely limited (3). The long-term consequences of SCI extend beyond physical disability, including reduced quality of life, increased risk of secondary complications, and substantial socioeconomic burden. The global incidence of traumatic spinal cord injury (SCI) in 2007 was estimated at 23 cases per million (179,312 cases per year), with 30% of these affected by tetraplegia (2).

The concept of modulating neural circuits to restore or modify physiological function has been a focus of scientific inquiry for centuries (4). Recent advances in neurophysiology have demonstrated that the spinal cord contains intrinsic locomotor networks capable of generating coordinated motor patterns even in the absence of supraspinal input. Experimental studies in animal models and humans have shown that these spinal circuits may remain functionally viable after injury and may be reactivated through neuromodulation. Epidural spinal cord stimulation (eSCS) is one such approach, delivering electrical stimulation to the dorsal surface of the spinal cord to activate afferent fibers and spinal interneuronal networks that can facilitate the re-engagement of dormant motor circuits. In clinical practice, epidural electrical stimulation has often been delivered using neuromodulation devices originally developed for the treatment of chronic pain (5).

.Over the past decade, several pioneering clinical studies have demonstrated the therapeutic potential of epidural stimulation for individuals with chronic SCI (6–11). Angeli et al. (6, 12) reported that epidural stimulation combined with intensive rehabilitation enabled participants with chronic motor-complete injuries to achieve standing and voluntary movements. Darrow et al. (8, 13) further demonstrated that targeted stimulation of spinal networks could evoke volitional motor responses below the level of injury, and that sustained SCS may enable emergence of voluntary movement even in the absence of active stimulation. More recently, Courtine et al. (5, 14) developed spatiotemporally patterned stimulation paradigms that enabled individuals with chronic SCI to regain overground walking when combined intensive rehabilitation training. Collectively, these studies suggest that neuromodulation can facilitate meaningful functional recovery even years after injury. Increasing evidence also suggests that activity dependent reorganization of neuronal circuits across the brain-spinal cord axis may play a key role in functional recovery following neuromodulation.

Despite these promising advances, several challenges remain (15–17). Many prior studies have relied on specialized stimulation systems or highly individualized stimulation protocols that may limit scalability and broader clinical implementation. In addition, the neurophysiological mechanisms underlying recovery remain incompletely understood (18–21). Understanding how spinal and supraspinal networks re-organize during rehabilitation, and identifying biomarkers that predict neurological recovery, are essential steps toward developing next-generation adaptive neuromodulation systems (4, 14, 22, 23).

The RESTORES trial (RESToration Of Rehabilitative function with Epidural spinal Stimulation) was designed to address these challenges through a multimodal translational approach. The study combines epidural spinal cord stimulation using commercially available neuromodulation devices with structured neurorehabilitation, robotic-assisted locomotor training (19, 24, 25), and mental imagery techniques (26–28). In addition to evaluating clinical outcomes, the trial incorporates advanced neurophysiological monitoring—including surface electromyography (sEMG) (29, 30), electroencephalography (EEG) (31–33), resting-state functional magnetic resonance imaging (rs-fMRI) (34–36), and computer vision-based motion analysis (37, 38). These complementary modalities aim to characterize neural recovery and elucidate the mechanisms underlying functional improvement.

By integrating neuromodulation, advanced rehabilitation strategies and multimodal neurophysiological monitoring, this study seeks to advance the understanding of brain–spinal cord network plasticity following chronic SCI. Insights gained from this work may inform the development of adaptive closed-loop neuromodulation systems capable of dynamically adjusting stimulation parameters in response to neural activity and motor intent; ultimately improving therapeutic outcomes for individuals living with spinal cord injury.

## 2 Methods and analysis

### 2.1 Trial design

The RESTORES trial is a single-center, prospective, single-arm feasibility study conducted at the National Neuroscience Institute, Tan Tock Seng Hospital (TTSH), Singapore. The study will be conducted in accordance with the ethical principles of the Declaration of Helsinki and the International Council for Harmonisation Good Clinical Practice (ICH-GCP) guidelines. The RESTORES trial is registered with ClinicalTrials.gov (Identifier: NCT05644171). Registration was completed prior to the enrolment of the first participant. The registry includes all items of the WHO Trial Registration Data Set. The full record is available at: https://clinicaltrials.gov/ct2/show/NCT05644171. The protocol version reported in this manuscript is Version 7 dated 17 Dec 2025. This study protocol is reported in accordance with the Standard Protocol Items: Recommendations for Interventional Trials (SPIRIT) guidelines. The SPIRIT checklist is provided as a supplementary file (39). The schedule of enrolment, interventions and assessments is presented in a SPIRIT figure (Figure 1; Table 1).

**Figure 1:**
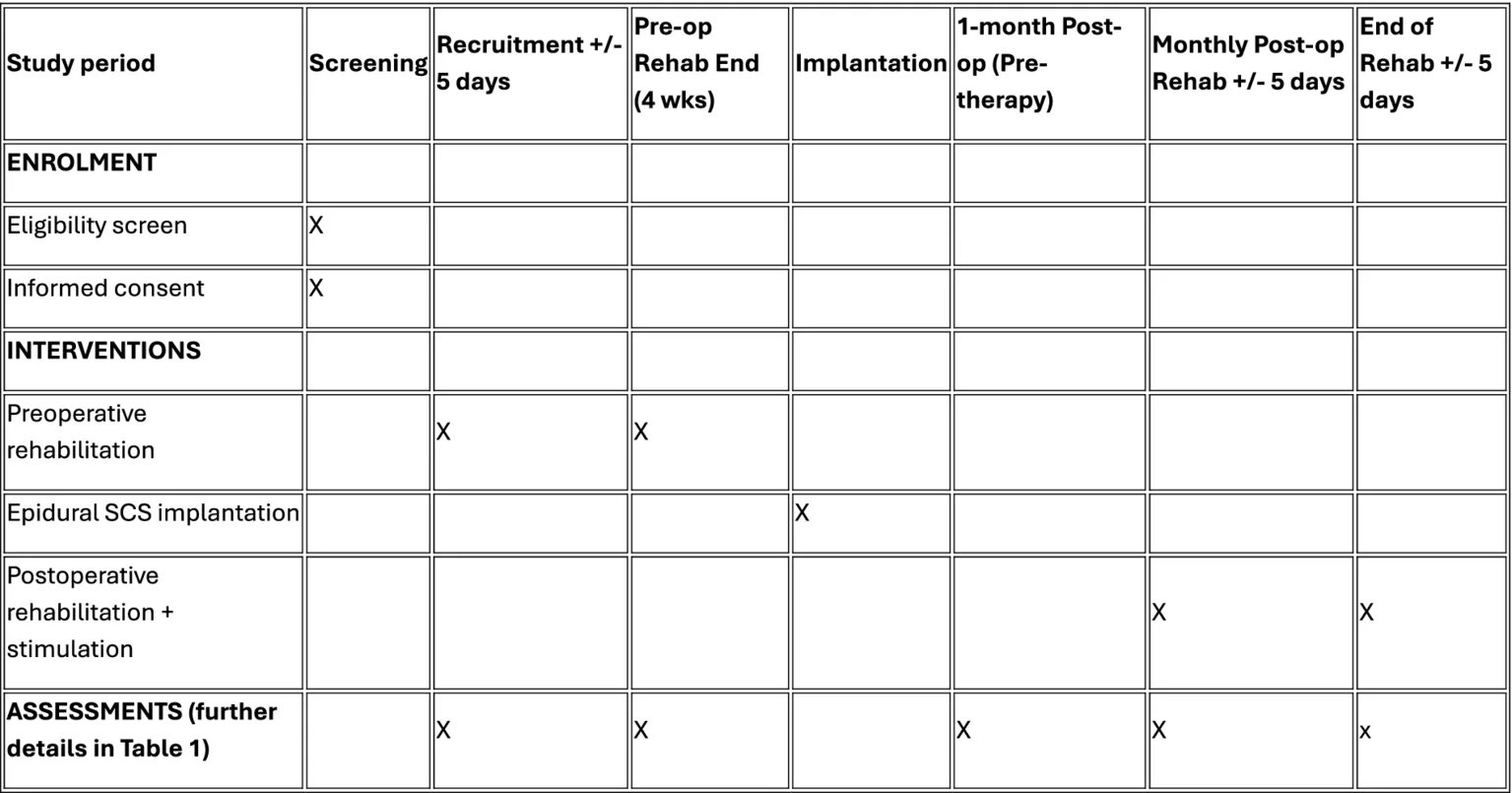
SPIRIT schedule of enrolment, interventions, and assessments for the RESTORES trial. This figure summarizes the timing of participant enrolment, epidural spinal cord stimulation implantation, rehabilitation interventions, and outcome assessments in accordance with SPIRIT recommendations.

**Table 1:** Schedule of assessments performed during the study’s process assessments beyond the 24-week intensive rehabilitation period (safety and device-integrity follow-up at 12, 18 and 24 months post-implantation) are described in the text.

	At recruitment ± 5 Days	End of rehabilitation training (4 weeks) ± 5 Days	1 month post-operative prior to onset of therapy	Once every month of post-operative rehabilitation training and stimulation ± 5 Days	End of intensive rehabilitation training ± 5 Days
Neurological assessments					
AIS scale	X	X	X	X	X
Spinal cord independence measure	X	X	X	X	X
Walking index for spinal cord injury II scale	X	X	X	X	X
Truncal assessment scale	X	X	X	X	X
5x sit to stand test	X	X	X	X	X
10-metre walk test	X	X	X	X	X
2-min walk test	X	X	X	X	X
Dynamometer for grip strength	X	X	X	X	X
Peak cough flow	X	X			X
Autonomic function assessments					
Bladder assessment	X	X	X	X	X
Bowel assessment	X	X	X	X	X
Quality of life assessments					
WHO quality of life questionnaire	X		X		X
	At recruitment ± 5 Days	End of rehabilitation training (weeks) ± 5 Days	1 month post operative prior to onset of therapy	Every week of post-operative rehabilitation training and stimulation ± 5 Days	End of intensive rehabilitation training ± 5 days
Neurophysiological assessments					
sEMG signals	X	X	X	X	X
EEG signals and goniometer signals	X	X	X		X
Resting state functional magnetic resonance imaging (rs-fMRI)	X				X

AIS, American Spinal Injury Association score; fMRI, functional magnetic resonance imaging; EEG, Electroencephalography; SCIM, Spinal Cord Independence Measures; sEMG, surface electromyography; TASS, Trunk Assessment for Spinal cord injury; WISCI II, Walking Index of Spinal Cord Injury II.

### 2.2 Approval committee

Ethical approval for the study has been obtained from the SingHealth Institutional Review Board and the National Healthcare Group Institutional Review Board prior to study initiation.

### 2.3 Human ethics approval and consent to participate

Written informed consent will be obtained from all participants before any study-specific procedures are performed. Participant confidentiality will be maintained using coded identifiers and secure storage of research data. The findings of this study will be disseminated through peer-reviewed publications, scientific conferences, and academic presentations, with the aim of advancing neuromodulation-based therapies for individuals with spinal cord injury.

### 2.4 Patient and public involvement

Patients and the public were not involved in the design, conduct, reporting or dissemination plans of our research.

### 2.5 Participant population and recruitment

The study population will consist of adults with chronic spinal cord injury (SCI) who are medically stable and able to safely participate in a rehabilitation-based neuromodulation protocol. Participants must be aged 21 years or older and have a chronic injury of more than 1 year in duration. Eligible participants must have a neurological level of injury between C5 and T12 and be classified as American Spinal Injury Association Impairment Scale (AIS) grade A or B. Participants are required to demonstrate preserved segmental reflexes below the level of injury on neurological examination. These criteria are intended to ensure that participants have stable chronic injuries and preserved spinal circuitry below the lesion that may be responsive to neuromodulatory interventions. In addition, participants must have sufficient physical and cognitive capacity to participate in the structured rehabilitation program required by the study protocol.

Participants will be excluded if they have medical conditions that would significantly increase the risk associated with study participation or interfere with rehabilitation. Key exclusion criteria include severe medical comorbidities, severe dysautonomia, or musculoskeletal conditions that would limit safe participation in training or assessments, including severe spasticity, contractures, fractures, pressure sores or severe osteoporosis. Participants with active psychiatric illness or substance abuse that may impair adherence to study procedures will also be excluded. Additional exclusion criteria include pregnancy or lactation and the presence of progressive spinal disease.

### 2.6 Device

Participants are implanted with a Medtronic Intellis™ AdaptivStim neurostimulator (model 97,719) connected to a Specify SureScan MRI paddle lead. The electrode configuration and implantation level will vary according to the neurological level of injury. Participants with tetraplegia will undergo implantation of a paddle lead with a 8–8 configuration positioned in the cervical spinal region to target upper limb and cervical spinal circuits. Participants with paraplegia will undergo implantation of a paddle lead with a 5–6-5 electrode configuration positioned over the relevant spinal segments to target lumbosacral motor networks.

The stimulator settings and electrode configurations for each participant may be adjusted according to the experimental protocol and physiological responses observed during intraoperative testing and postoperative programming.

## 3 Study procedures

### 3.1 Screening

Participant recruitment (40) will be conducted through a coordinated national referral network involving clinicians and rehabilitation centers specializing in spinal cord injury (SCI) care across Singapore. The study team will collaborate closely with referring physicians within this network to identify potential participants. Clear inclusion and exclusion criteria will be shared with participating clinicians to facilitate preliminary identification and pre-screening of suitable candidates prior to referral. This approach leverages Singapore’s well-integrated SCI rehabilitation network and helps ensure that individuals referred to the study team are likely to meet eligibility requirements, thereby minimizing unnecessary screening and improving recruitment efficiency.

Potential participants identified by referring clinicians will be referred to the study team at the National Neuroscience Institute for formal screening. To further enhance recruitment and outreach, the study team will engage with national and regional SCI organizations, rehabilitation centers, and patient support groups to disseminate information about the trial through community outreach initiatives, educational sessions, print materials, and digital platforms. These partnerships are intended to increase awareness of the study within the SCI community while providing a trusted pathway for communication between potential participants and the research team. Eligible individuals will be invited to attend a screening visit at the study center, during which they will receive detailed information regarding the study procedures, risks, and potential benefits. Written informed consent will be obtained prior to the initiation of any study-specific procedures.

Following consent, participants will undergo comprehensive screening assessments to confirm eligibility for enrolment. These assessments will include review of medical history, neurological examination, and confirmation of spinal cord injury characteristics, including neurological level of injury and American Spinal Injury Association (AIS) classification. Baseline spinal imaging will be reviewed to verify the anatomical level of injury and assess suitability for epidural electrode implantation. Participants will also be evaluated for medical comorbidities that may increase perioperative risk, as well as for dysautonomia and musculoskeletal conditions that could interfere with safe participation in the rehabilitation program.

Participants who meet all eligibility criteria will be enrolled into the study. Individuals who withdraw prior to device implantation will be replaced, while participants who withdraw after implantation will be considered lost to follow-up; however, data collected up to the point of withdrawal will be retained unless the participant requests otherwise (Figure 2).

**Figure 2:**
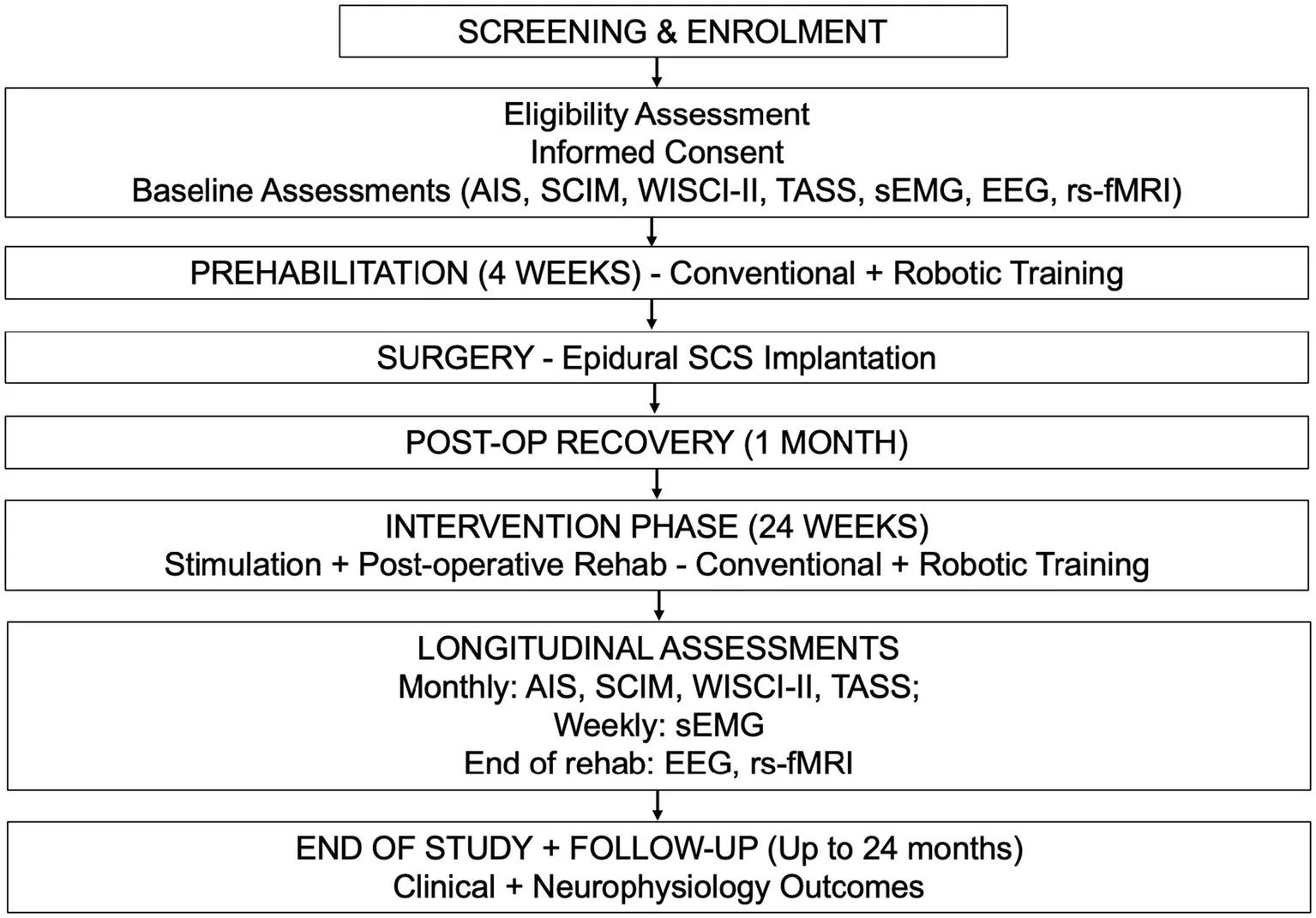
Study schema.

### 3.2 Baseline assessments

Baseline demographic data and clinical assessments will be obtained following enrolment. Neurological function will be assessed using the American Spinal Injury Association (AIS) scale and functional outcome measures including the Spinal Cord Independence Measure version III (SCIM-III), Walking Index for Spinal Cord Injury II (WISCI-II), Trunk Assessment Scale for spinal cord injury (TASS), and standardized mobility assessments. Additional baseline measurements will include respiratory assessment with peak cough flow, grip strength using dynamometry, and autonomic function assessments including bladder and bowel function.

Baseline neurophysiological recordings will be obtained using surface electromyography (sEMG) from major upper and lower limb muscle groups. Concurrent electroencephalography (EEG)-Goniometer recordings and resting-state functional MRI (rs-fMRI) will also be performed prior to implantation to characterize baseline neural activity and connectivity patterns. These baseline measurements will serve as reference values for longitudinal comparison following epidural stimulation and rehabilitation.

Participants will undergo a four-week period of preoperative rehabilitation to improve conditioning and verticalization tolerance. Training sessions of one hour duration will occur twice weekly with one conventional physiotherapy and one robot-assisted gait training session. The conventional physiotherapy includes stretching and strengthening exercises of the trunk and limbs, supported standing training, and cycling exercises to maintain joint mobility and muscle conditioning. Participants will use the robotic exoskeleton device, EksoGT, (Ekso Bionics, Richmond, California, USA), during this phase in preparation for postoperative rehabilitation training.

### 3.3 Study’s primary and secondary endpoints

#### 3.3.1 Primary endpoint

Assessing the safety of SCS implant in participants with chronic SCI.

Given the off-label usage of the device, safety assessments will include monitoring adverse events (AEs), serious adverse events (SAEs), and device retention throughout the study. Events will be categorized based on their severity and relationship to the intervention. All safety data will be regularly reported to the Ethics Committee and an independent Data and safety monitoring board (DSMB). The DSMB will review cumulative safety data to ensure participant well-being and provide oversight.

Success will be defined as the <10% device removal for safety reasons during the 6-month post- implant evaluation period. With a planned sample of 15 participants, this threshold corresponds to no more than one participant undergoing device explantation for safety reasons (i.e., ≤1 of 15); the primary safety endpoint is therefore considered met if safety-related device explantation occurs in one or fewer participants.

#### 3.3.2 Secondary endpoints

The secondary endpoints include the following:

(1) Achieve an *improvement in neurological function*, defined as the restoration of volitional muscle control below the level of injury. This will be measured by an improvement in the AIS motor score in 15 participants with chronic motor-complete SCI, including the ability to achieve independent functional overground walking.

The AIS motor scale (41), a widely used tool for assessing SCI, provides a quantitative measure of disability and the neurological level of injury by evaluating motor and sensory functions in both the upper and lower limbs. Progress will be objectively tracked at scheduled time points (Figure 1; Table 1).

Success will be defined as a minimum improvement of 4 points (AIS score) for minimally clinically important difference (MCID) and/or the attainment of independent walking with or without aids or orthoses. AIS motor scores will be analyzed both as the total motor score and as separate upper-extremity (UEMS) and lower-extremity (LEMS) motor subscores, and will be reported separately for the tetraplegic (cervical implant) and paraplegic (thoraco-lumbar implant) cohorts, reflecting the different motor pools targeted at each implantation level. Limb-relevant secondary measures are interpreted accordingly: grip strength and peak cough flow are analyzed primarily in the cervical/tetraplegic cohort, and lower-limb and gait measures primarily in the paraplegic cohort. Attainment of independent overground walking, with or without aids or orthoses, will be reported separately. Given the small sample size, these subgroup analyses are considered exploratory and hypothesis-generating.

(2) Improvement in *functional outcomes measures*.

Improvement in functional outcomes will be objectively tracked at scheduled time points (Figure 1; Table 1) using the following validated functional scales:

i. Spinal cord independence measure (SCIM): SCIM is a validated disability scale that evaluates performance in activities of daily living and mobility across three principal domains: self-care (sub score: 0–20), respiration and sphincter management (0–40), and mobility (0–40). Each domain is weighted according to its proportional contribution to the patient’s overall activity, with a total score ranging from 0 to 100, where higher scores indicate greater independence (42). Success is defined as a minimum improvement of 4 points for MCID (43).

ii. Walking index of Spinal Cord Injury II (WISCI II): WISCI II is a reliable functional scale for measuring walking improvements in SCI patients. It assesses impairment severity and assistance needed to walk 10 meters, with scores ranging from 0 (most impaired) to 20 (least impaired), based on assistive device use and physical support (44). Success is defined as a minimum improvement of 3 points for MCID (45).

iii. Trunk assessment scale for spinal cord injury (TASS): Truncal stability is essential for standing, walking, and voluntary movements. The TASS, a validated 9-item scale with high intra- and inter-rater reliability, effectively assesses truncal balance in SCI patients (46). A change of at least 3 WISCI-II levels will be used as a clinically meaningful benchmark where the scale is responsive; floor effects at baseline will be explicitly considered (41, 45).

(3) Improvement in *autonomic (bladder and bowel)* neurological function. This will be objectively tracked with standardized questionnaires (Neurogenic Bladder Symptom score and Neurogenic Bowel Dysfunction score respectively) according to the scheduled time points (Figure 1; Table 1). Although micturition and defaecation are governed primarily by thoraco-lumbar sympathetic and sacral parasympathetic circuits, improvements in bladder and bowel function have been reported following epidural stimulation at both lumbosacral and more rostral levels, attributed to activity-dependent reorganization across multi-segmental spinal and supraspinal autonomic pathways and to the effects of intensive activity-based training (4, 13). Autonomic outcomes will therefore be examined across the whole cohort, with cervically implanted participants analyzed as an exploratory subgroup. To complement the symptom questionnaires, post-void residual volume (by bladder ultrasound scan) and voiding/bowel diaries will be collected at the scheduled time points; formal urodynamic studies are not mandated in this feasibility protocol and this is acknowledged as a limitation.
  Success will be defined as meaningful reduction in the Neurogenic Bladder Symptom Score and/or Neurogenic Bowel Dysfunction score at the individual-participant level. As validated point-based MCIDs for these instruments are not well established, the autonomic endpoint is reported descriptively and treated as exploratory rather than as a formal success threshold.
(4) Improvement in our recruited participants’ *quality of life*. This will be assessed using standardized questionnaires (WHO Quality of Life) according to the scheduled time points (Figure 1; Table 1). The WHOQOL evaluates physical health, psychological health, social relationships, and environmental factors. Responses will be scored on a Likert scale, aggregated into domain-specific scores, and standardized to a 0–100 scale for comparison. Success will be defined as a minimum improvement of 5 points for MCID.
(5) Utilize surrogate sEMG signals, computer vision techniques and rsFMRI analyses to model recovery of neural connections and deepen our understanding of brain-spine neuroplasticity. These insights will inform the development of closed-loop technology systems for personalized and adaptive spinal cord stimulation. [Further details below]

### 3.4 Stimulator implantation technique and intraoperative mapping

Epidural spinal cord stimulation electrodes will be implanted using a standard neurosurgical technique (7). Under general anesthesia, a limited laminectomy will be performed to expose the epidural space at the target spinal level. The electrode paddle will be placed on the dorsal surface of the dura overlying the spinal cord. The exact level of implantation will be determined based on the participant’s neurological level of injury and preoperative imaging.

Intraoperative neurophysiological monitoring will be performed using surface electromyography recordings from multiple muscle groups. Stimulation will be delivered through the electrode contacts while monitoring muscle responses to confirm recruitment of the targeted motor pools. Electrode position may be adjusted rostrally or caudally to maximize activation of relevant muscle groups based on observed electromyographic responses. Intraoperative test stimulation is delivered through the paddle contacts as biphasic, actively charge-balanced pulses at 2 Hz, 350 μs pulse width, and 0–10 mA, consistent with the mapping protocol established in our pilot study (7). Contacts are operated in a bipolar configuration and are systematically selected to evoke posterior root-muscle reflex responses (PRMRRs) in the targeted myotomes, monitored by surrogate surface EMG. In the paraplegic (thoraco-lumbar implant) cohort, recordings are obtained from rectus femoris, vastus lateralis, medial hamstring, tibialis anterior, medial gastrocnemius, and soleus; in the tetraplegic (cervical implant) cohort, from deltoid, brachialis, triceps brachii, extensor carpi radialis longus, flexor digitorum profundus, opponens pollicis, abductor pollicis longus, and extensor digitorum. Optimal positioning is defined *a priori* as placement of the paddle at the midline that produces symmetrical, segmentally appropriate PRMRRs across the targeted myotomes at the lowest stimulation threshold — corresponding, in the pilot, to equal bilateral L1–L5 and S1 activation for thoraco-lumbar placement, and to equal bilateral C5–T1 activation for cervical placement.

Once optimal positioning is confirmed, the electrode will be secured in place using surgical adhesives or anchoring techniques. The electrode lead will then be tunneled subcutaneously and connected to an implantable pulse generator placed in a subcutaneous pocket. Following device implantation, surgical wounds will be closed in layers. A schematic overview of the intervention, including epidural electrode placement on the dorsal surface of the spinal cord, is provided in Figure 3.

**Figure 3:**
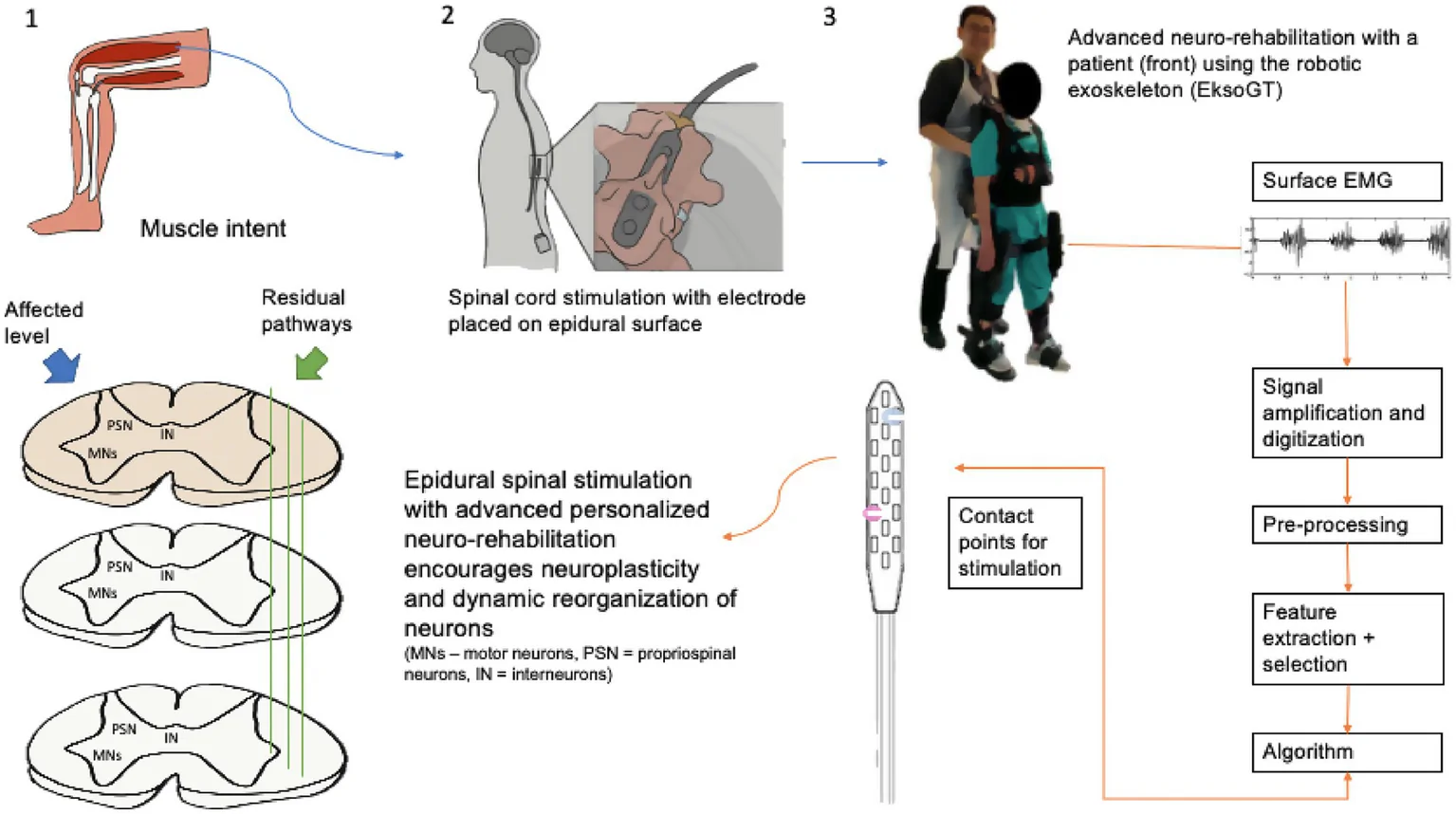
Overview of the RESTORES multimodal intervention. (1) Volitional muscle intent below the level of injury; (2) epidural spinal cord stimulation delivered via a paddle electrode placed on the dorsal epidural surface of the spinal cord (contact points for stimulation indicated), with spinal cord cross-sections showing the affected level, residual pathways, and motor neuron (MN), propriospinal neuron (PSN), and interneuron (IN) networks; and (3) personalized robotic exoskeleton (EksoGT) neurorehabilitation. Surface electromyography drives the signal-processing pipeline (amplification and digitization, pre-processing, feature extraction and selection, and decoding algorithm) used to characterize recovery and inform closed-loop stimulation.

Participants will be monitored postoperatively in the intensive care unit for at least 24 h before transfer to the general ward and subsequent discharge once medically stable.

### 3.5 Perioperative rehabilitation and follow-up

Stimulation will be activated approximately one month following implantation to allow for adequate wound healing. Activation of the stimulator will coincide with the commencement of postoperative rehabilitation training.

Participants will undergo intensive rehabilitation consisting of one-hour training sessions five days per week over a 24-week period (47). Rehabilitation will include conventional physiotherapy combined with robotic exoskeleton-assisted gait training. All training sessions will be performed with epidural stimulation to promote activity-dependent neuroplasticity within spinal networks. Each rehabilitation session also incorporates a structured motor-imagery component of approximately 10–15 min, in which participants perform cued kinaesthetic imagery of the trained movement (for example, stepping or grasp) immediately before and during the corresponding physical or robot-assisted task, on each of the five training days per week across the 24-week period. Surface electromyography recordings will be obtained during rehabilitation sessions both with and without stimulation to evaluate recruitment of muscles below the level of injury. Intentional movement attempts and actual movements will be recorded and analyzed in relation to the recorded sEMG signals. Stimulation parameters including amplitude, pulse width, frequency, and stimulation configuration may be adjusted based on observed neurophysiological responses and clinical progress. Parameter adjustment is performed by the study neurosurgeon/physician, in consultation with the treating physiotherapist. Programming is reviewed at the start of the postoperative rehabilitation phase and thereafter at least weekly during the training period, and additionally whenever sEMG responses, tolerability, or functional performance indicate a need. Amplitude is titrated to the highest level producing functional motor recruitment below the sensory-discomfort and pain thresholds, with pulse width, frequency, and active-contact configuration selected to optimize task-specific recruitment of the targeted motor pools. For rhythmic stepping, interleaving programs delivering approximately 50 Hz, 100–400 microseconds pulse width, and 5–20 mA are used, following the approach established in our pilot study (7).

Participants will undergo repeated neurological, respiratory, autonomic, and functional assessments at predetermined intervals throughout the study period. Long-term follow-up will be provided by the neurosurgical and rehabilitation teams for up to 24 months after implantation. Table 1 details the assessment schedule through the 24-week intensive rehabilitation period. Beyond this period, participants attend safety and device-integrity follow-up visits at 12, 18 and 24 months post-implantation, at which adverse events, device status, and AIS and functional status are recorded.

### 3.6 Additional tests and computational analysis

Additional neurophysiological and neuroimaging assessments will be performed throughout the study to characterize neural recovery and guide optimization of stimulation parameters. Electroencephalography recordings will be used to evaluate cortical activity associated with motor intent and attempted movements. Resting-state functional magnetic resonance imaging will be performed at defined timepoints to assess functional connectivity within brain networks involved in motor control and to investigate experience-dependent plasticity within the brain-spinal cord axis.

EEG is recorded both with stimulation OFF and during active stimulation. For recordings acquired during stimulation, stimulation artefact is managed using hardware synchronization of stimulation timing, template subtraction/blanking of stimulus pulses, band-pass and notch filtering, and independent component analysis for residual-artefact rejection. EEG is acquired synchronously with joint goniometer signals; in this EEG-goniometer framework, continuous joint-angle traces are time-locked to the EEG to label movement onset and phase, enabling analysis of movement-related cortical dynamics and decoding of gait- and movement-intent from the EEG (48, 49).

Resting-state imaging (35, 50) will be acquired using a 1.5-Tesla MRI scanner equipped with a multi-channel head coil. All MRI acquisitions are performed with the neurostimulator switched OFF and in MRI-conditional mode, in accordance with the manufacturer’s SureScan MRI conditions, so that imaging reflects stimulation-independent network organization and avoids stimulation-related artefact. Participants will undergo anatomical imaging followed by resting-state scans while maintaining visual fixation on a central crosshair. Task-based paradigms involving motor imagery of limb movement will also be performed to evaluate task-related brain activation. The motor-imagery paradigm uses a block design (alternating 20-s imagery and 20-s rest blocks over runs of approximately 6 min). Participants perform cued kinaesthetic (first-person) motor imagery of rhythmic lower-limb stepping and, for cervically injured participants, of hand grasp., while avoiding overt movement.

Surface electromyography data collected during rehabilitation will be analyzed using advanced computational approaches to decode movement intentions (30, 51) and characterize the evolution of neural control signals during recovery (30, 52). Computer vision techniques (38) will also be applied to video recordings of rehabilitation sessions to quantify kinematic features of movement and gait patterns. These multimodal datasets will be integrated using machine learning methods to develop predictive models of motor recovery and to inform future development of closed-loop neuromodulation systems. Computer-vision movement analysis uses markerless pose estimation applied to synchronized video of rehabilitation and assessment sessions. Two calibrated RGB cameras are positioned in the sagittal and frontal planes at fixed height and distance relative to the training platform; recordings are processed with an open-source markerless pose-estimation pipeline to extract joint keypoints and derive kinematic features, including joint angles, step and stride parameters, and inter-limb coordination.

### 3.7 Trial governance and oversight

The RESTORES trial is coordinated by the National Neuroscience Institute (NNI), Singapore, which serves as the coordinating center responsible for overall study conduct, protocol implementation, and regulatory compliance. The principal investigator oversees trial execution, participant recruitment, and clinical management across participating departments.

A multidisciplinary steering committee comprising investigators from neurosurgery, rehabilitation medicine, biomedical engineering, and data science provides strategic oversight of study progress, protocol adherence, and scientific direction. The steering committee meets periodically to review recruitment, protocol deviations, and operational issues.

An independent Data Safety Monitoring Board (DSMB), consisting of experts in neuromodulation, spinal cord injury, and clinical trials, provides external oversight of participant safety and study conduct. The DSMB reviews adverse events, serious adverse events, and safety outcomes at predefined intervals and makes recommendations regarding trial continuation.

Data management is coordinated by the study data management team at NNI in collaboration with institutional research support units. The team is responsible for database design, data entry procedures, quality checks, and secure storage of de-identified participant data in accordance with institutional policies.

Endpoint assessment is performed by trained investigators using standardized outcome measures. Where applicable, assessments are conducted by personnel independent of the surgical team to minimize bias.

The study team also includes rehabilitation therapists, neurophysiology engineers, and research coordinators who support participant assessments, data acquisition, and intervention delivery. All personnel involved in the trial receive protocol-specific training prior to participation.

## 4 Statistical analysis

This study is designed as a single-arm Phase II feasibility trial enrolling 15 participants with chronic motor-complete spinal cord injury. The sample size of 15 participants is considered appropriate for a feasibility study to evaluate safety, protocol adherence, and preliminary signals of efficacy. Descriptive statistics will be used to summarize baseline demographic and clinical characteristics. Continuous variables will be reported as mean ± standard deviation or median with interquartile range, depending on distribution. Categorical variables will be reported as frequencies and percentages. Changes in neurological and functional outcomes over time will be analyzed using repeated-measures analysis of variance (ANOVA) or linear mixed-effects models, depending on data distribution and missing data patterns. Neurophysiological datasets including sEMG, EEG, and rs-fMRI will undergo computational signal processing and network analyses to characterize neural activity patterns and their relationship to functional recovery. All statistical analyses will be performed using R statistical software. Safety outcomes will be summarized descriptively, including incidence and type of adverse events. As this is a single-arm feasibility study, all secondary and neurophysiological endpoints are exploratory: they are reported with point estimates and 95% confidence intervals without formal correction for multiple comparisons, and any *p*-values are interpreted descriptively rather than as confirmatory hypothesis tests. Subgroup analyses by implant level (cervical vs. thoraco-lumbar) and by AIS grade are likewise exploratory and hypothesis-generating. The 5-point quality-of-life reference denotes a change on the 0–100 standardized WHOQOL domain scale that we regard as the smallest clinically meaningful difference; as a validated MCID for the WHOQOL in chronic SCI is not well established, this value is used descriptively rather than as a formal success criterion.

## 5 Data and safety monitoring

An independent Data Safety Monitoring Board (DSMB) will oversee the safety and integrity of the trial.

The DSMB will consist of independent experts in neuromodulation, spinal cord injury, and clinical trial methodology who have no financial or scientific conflicts of interest related to the study.

The DSMB will periodically review:

- Study progress and protocol adherence
- Participant safety and adverse event reporting
- Data quality and trial conduct

Serious adverse events will be reported immediately to the DSMB and institutional review boards in accordance with regulatory requirements. All study data will be stored in secure institutional databases with restricted access. Participant data will be coded using unique study identifiers to maintain confidentiality.

## 6 Discussion

Chronic motor-complete spinal cord injury has historically been considered a condition with limited potential for neurological recovery (53, 54). Conventional rehabilitation strategies have therefore focused primarily on preventing complications and optimizing functional independence through compensatory approaches rather than restoring neural function. However, an expanding body of experimental and clinical evidence (6, 8, 12–14) together with findings from our group’s preliminary work (7, 47), suggests that the injured spinal cord retains a significant capacity for neuroplasticity even years after injury. In this context, neuromodulation—particularly epidural spinal cord stimulation—has emerged as a promising strategy for reactivating dormant spinal networks and facilitating functional recovery (16, 17, 55).

The RESTORES trial Phase II (7) builds upon this evolving paradigm by evaluating a multimodal therapeutic strategy (56) that integrates epidural spinal cord stimulation with personalized neurorehabilitation, robotic-assisted locomotor training, and motor imagery techniques. Importantly, unlike many prior studies that relied on experimental or highly customized stimulation systems, this trial utilizes commercially available neuromodulation technology. This design choice enhances the translational relevance and potential scalability of the intervention, supporting broader clinical adoption if efficacy is demonstrated.

A key innovation of this study lies in the integration of multimodal neurophysiological monitoring to elucidate the mechanisms underlying recovery. Surface electromyography provides real-time insights into motor pool recruitment and the evolution of voluntary muscle activation during rehabilitation. Concurrently, computer vision–based analysis enables objective quantification of movement kinematics, including joint coordination and gait symmetry. In parallel, electroencephalography and resting-state functional MRI facilitate characterization of large-scale cortical network reorganization and the relationship between cortical activity and spinal motor output.

Recent advances in deep neural network architectures (48, 49) have demonstrated the feasibility of decoding gait-related neural signals from EEG in healthy individuals, suggesting that neural signatures of motor intent can be robustly extracted. The integration of EEG, sEMG, imaging, and computer vision data within this study provides a unique opportunity to examine how neural activity evolves across the brain–spinal cord axis during rehabilitation. Such multimodal datasets may enable the identification of biomarkers of recovery and the development of predictive models of treatment response. Ultimately, these insights could inform the design of adaptive, closed-loop neuromodulation systems capable of dynamically adjusting stimulation parameters based on neural signals or movement intent (9, 57).

This study has several limitations. As a single-arm Phase II feasibility trial with a relatively small sample size, it is not powered to establish definitive treatment efficacy. The absence of a control group limits causal inference, and placebo or expectation effects associated with intensive rehabilitation cannot be fully excluded. In addition, heterogeneity in injury characteristics and chronicity may influence individual responses to therapy. However, these limitations are inherent to early-phase neuromodulation studies and are balanced by the study’s focus on safety, feasibility, and mechanistic understanding. The incorporation of detailed phenotyping and longitudinal multimodal neurophysiological assessments aims to better characterize variability in recovery trajectories and provide objective measures that complement clinical outcomes. Specifically, because all training is delivered with stimulation switched ON, the design cannot on its own separate the effects of epidural stimulation from those of intensive activity-based rehabilitation. This confound is partially mitigated by within-participant comparisons of neurophysiological and functional responses recorded with stimulation ON versus OFF (for example, sEMG recruitment during rehabilitation and stimulation-OFF assessments), which provide an internal reference for stimulation-specific effects; definitive separation of these contributions will nonetheless require a future controlled trial incorporating a sham- or delayed-stimulation arm. Several outcome measures are also subject to floor effects in this motor-complete (AIS A/B) population: in particular, the WISCI-II is expected to be at or near its floor (score 0) at baseline in most participants, which limits its sensitivity to detect the 3-point MCID unless participants regain assisted stepping, so it is most informative in the subset who progress to overground stepping.

The decision not to include a concurrent control group reflects the feasibility and safety objectives of this first-in-center Phase II study, together with practical and ethical considerations specific to invasive neuromodulation. Sham implantation would expose participants to the risks of surgery and general anesthesia without therapeutic intent and was therefore considered difficult to justify ethically at this stage, while the small sample size and marked heterogeneity of chronic motor-complete SCI limit the value of between-group randomization in a feasibility cohort. Instead, each participant serves as their own longitudinal control through repeated pre- and post-implantation assessments and within-session stimulation ON/OFF comparisons. The resulting safety, feasibility, and mechanistic data - including effect-size estimates and variability across the multimodal outcomes - are intended specifically to inform the design and powering of a subsequent multicenter randomized controlled trial incorporating a sham- or delayed-stimulation comparator, blinded outcome assessment, and stratification by injury level. Future work will further focus on translating the multimodal neurophysiological biomarkers identified here into closed-loop control signals for adaptive stimulation, and on defining responder phenotypes to guide patient selection. Despite these constraints, the RESTORES trial offers a comprehensive framework that bridges mechanistic neuroscience and clinical translation. By integrating commercially available neuromodulation technology with advanced rehabilitation and computational modeling, this study is positioned to generate critical insights into the neural basis of recovery following chronic SCI. The findings will inform the design of future multicenter randomized controlled trials and contribute to the development of next-generation neuromodulation strategies.

Beyond evaluating clinical outcomes, the multimodal datasets generated in this trial may provide an unprecedented opportunity to characterize the dynamic interactions between cortical, spinal, and musculoskeletal systems during recovery. If successful, this work may contribute to a paradigm shift in the treatment of spinal cord injury—challenging the long-standing assumption of irreversibility in chronic motor-complete SCI and advancing the development of personalized, adaptive neuromodulation therapies aimed at restoring meaningful neurological function.
